# The micro-macro interplay of economic factors in late-life loneliness: Evidence from Europe and China

**DOI:** 10.3389/fpubh.2022.968411

**Published:** 2022-09-13

**Authors:** Jing Wu, Jing Zhang, Tineke Fokkema

**Affiliations:** ^1^Department of Sociology and Work Science, University of Gothenburg, Gothenburg, Sweden; ^2^Department of Public Administration and Sociology, Erasmus University Rotterdam, Rotterdam, Netherlands; ^3^Netherlands Interdisciplinary Demographic Institute (NIDI)-KNAW/University of Groningen, The Hague, Netherlands

**Keywords:** income inequality, welfare generosity, loneliness, older people, Europe, China, socioeconomic status

## Abstract

Individual socioeconomic status has a significant impact on whether older adults can initiate and maintain social relationships and participate in society, hence it affects loneliness. At the macro level, income inequality is expected to increase the risk of loneliness by eroding social cohesion and trust, while welfare generosity might protect people from loneliness. The aim of the study is to explore whether income inequality and welfare generosity at the country level moderate the effect of socioeconomic status at the individual level on late-life loneliness. Data were obtained from the HRS family of surveys – the Survey of Health, Aging and Retirement in Europe (SHARE) (wave 5, 2011/12) and China Health and Retirement Longitudinal Study (CHARLS) (wave 2, 2012/13). Respondents aged 50 years and older from twelve European countries and China were included in the study. Logistic country fixed effect models were used in the analysis. The findings show a stronger effect of individual socioeconomic status on late-life loneliness in more income-unequal societies and a weaker effect in more welfare-generous societies. There is a need to consider the impact of income distribution and welfare spending on the risk of loneliness among those older adults with low socioeconomic status when tailoring preventive programs and interventions to reduce loneliness among this vulnerable group.

## Introduction

Loneliness has been defined as the subjective feeling of a mismatch between desired and actual number of meaningful social contacts (1). As people age, their level of loneliness increases (2) because considerable changes in life circumstances, including bereavement and health decline, accompany old age (3–5). The prevalence of loneliness in old age varies geographically. In the European setting, cross-country comparative studies show that the prevalence of loneliness among older adults aged 60 and older generally is lowest in Northern European countries, followed by Southern European countries, and highest in Central and Eastern European countries (6, 7). Literature on China reports prevalence rates of loneliness similar to their peers in Central and Eastern Europe (8–11).

A large body of literature shows that individual-level characteristics such as age, gender, health, marital status, socioeconomic status, social participation, social support, and social networks are associated with older adults' loneliness (12–17). To explain these cross-country differences in late-life loneliness, many studies focus on differences in population composition between countries (6, 7, 18, 19). However, these studies invariably show that country variation in loneliness partially remains after controlling for compositional differences. This has led to an increasing awareness that loneliness is not an exclusively individual phenomenon, but is embedded in social, cultural, and material contexts (20, 21). In recent years, several researchers have taken up the gauntlet by investigating whether country-level characteristics [macro-level factors, such as societal individualism (22, 23), level of trust (24), and type of welfare regime (25)] and interactions between individual characteristics and country (micro-macro interplay) help to explain cross-country differences in late-life loneliness. Lykes and Kemmelmeier (22), for instance, examined whether the experience of older adults' loneliness is culturally related. They ranked European countries on Hofstede's index of collectivism-individualism (26), where being part of traditional kinship networks is more normative in collectivistic societies and personal choice in forming social relationships is more valued in individualistic societies. Besides higher levels of loneliness among older adults in collectivistic societies, findings show that less frequent family contact is more closely linked to loneliness in collectivistic societies, while less frequent contact with friends weighs more in individualistic societies. Zoutewelle-Terovan and Liefbroer (23), looking at the effect of non-normative life transitions (i.e., never and off-time occurrence of partnership and parenthood) on late-life loneliness in twelve European countries, examined whether cross-country variation in the size of the effects could be explained by country differences in familialism values and opinions on economic insecurity. Results show that only the effect of childlessness (compared to those who experienced having a child “on time”) was larger in countries where individuals felt more strongly attached to traditional, familialism values. Rapoliene and Aartsen (24) were interested in whether the higher prevalence of late-life loneliness in Eastern European post-totalitarian countries, compared to other European countries and Israel, can be ascribed to lower levels of trust in people (generalized trust) and in the system: although lower levels of both types of trust were observed, generalized trust and trust in the system did not appear to directly affect loneliness. Instead, a low level of generalized trust was found to lead to social disengagement, in turn increasing the likelihood of feeling lonely. A final example is the study of Nyqvist et al. (25), who explored the impact of state involvement in social welfare on late-life loneliness, directly and indirectly through individual resources (like living conditions and level of social integration). They divided 20 European countries into five welfare regimes: Nordic, Continental, Anglo-Saxon, Southern, and Eastern. The Nordic regime, known for its high extent of state involvement in social welfare, had the lowest proportion of older adults feeling lonely (some of the time, most of the time, almost all of the time), followed by the Continental and Anglo-Saxon regimes. Older adults living in the Nordic regime were less dependent on their own social resources for loneliness compared to regimes where loneliness was to a greater extent conditioned by family and other social ties. It remains unclear, however, whether these findings are actually linked to differences in level of state welfare provision, as dummies for type of regime instead of specific country-level factors (such as social welfare spending and benefit receipt) were included.

To the best of our knowledge, no research has yet been conducted on the extent to which country-level economic factors influence the effect of individual-level socioeconomic status on older adults' loneliness. This study aims to investigate this micro-macro interplay of economic factors. Specifically, we will examine the moderating effects of countries' income inequality and welfare generosity on the association between individual-level household income and loneliness in later life.

## Theoretical framework

### Individual-level socioeconomic status and loneliness

Empirical studies have repeatedly shown that older adults with lower socioeconomic statuses (captured by factors like low educational level, low income, residential dissatisfaction, and living in deprived neighborhoods) are more likely to be lonely (14, 20, 27–29). While there is virtually no literature directly linking socioeconomic status to the experience of loneliness, several theoretical pathways have been proposed through which low socioeconomic status increases the risk of loneliness. Nearly all pathways consider socioeconomic status as a distal factor affecting the more proximate conditions for people's ability to optimize and diversify social contacts, and in turn loneliness (6, 18, 20, 30). The most-often cited pathway is that individuals of low socioeconomic status have fewer financial resources for initiating and maintaining social relationships (18, 31): for example, sufficient income is needed to invite people into one's home for a drink or dinner, or to go on an outing together. Financial resources also allow people to fully participate in society by being able to afford memberships in clubs and organizations, transport costs, and leisure activities. Another explanation is that lower socioeconomic groups generally have poorer social skills and lower self-esteem, which makes them less confident and uninhibited in social interactions and hence less attractive to others (6, 32). One pathway suggests the enhanced risk of poor physical and/or mental health status or reduced functioning, which can lead to fewer social interactions and lower participation in the wider society, including employment, volunteering, and leisure and other social activities (6, 33–35). Last, there is the greater likelihood of living in a deprived neighborhood, where there is often an accumulation of physical and socioeconomic problems (unsafe public spaces, lack of social cohesion, low participation levels, high proportion of low-income households) resulting in restricted social interactions (36–39). In an English representative survey, Victor and Pikhartova (40) pointed out that older people living in the most deprived areas were more likely to experience loneliness than their counterparts living in the least deprived areas.

### Income inequality, welfare generosity and loneliness

Income inequality at the country level may increase the risk of loneliness by eroding social cohesion and trust (24, 41). In egalitarian societies where differences in social status, power, and wealth are less prominent, people are more likely to be cooperative and have harmonious social relationships with others, generating mutual trust; in less egalitarian societies, people are less caring of others and more likely to compete with each other and distrust others (42–44). The social distance between people is greater in less egalitarian societies and individuals' social connections with the outside world are looser, increasing the risk of loneliness (43, 45, 46).

A high level of state involvement in welfare provisions may protect individuals from loneliness (25). By making social services and benefits available to individuals, people in generous welfare states are less dependent on personal and social resources to establish relationships with others, which increases people's ability to be socially integrated (25, 47, 48). Such generous welfare support can help individuals obtain their desired social capital, social networks, and trust (48, 49), lowering the likelihood of loneliness.

### Impact of individual-level socioeconomic status on loneliness in the context of income inequality and welfare generosity

Income differences are not only about comparison of income itself, but more about differences in quality of life and the perception of its social fairness (44, 50, 51). In societies with high income inequality, people with low socioeconomic status are aware that the good and rich social life enjoyed by their counterparts from the upper classes is never within their means. Studies show that relative deprivation, a sense of having less than one feels entitled to, causes stress as well as feelings of exclusion and being less valued (20, 27, 52). This implies that, at the individual level, low socioeconomic status has a stronger effect on loneliness in a more income-unequal society. We hypothesize that older adults with a lower socioeconomic status in countries with less egalitarian income distribution are more likely to be lonely than their counterparts in countries with more egalitarian income distribution (Hypothesis 1).

A high level of state involvement in welfare provisions may particularly protect socioeconomically disadvantaged people from loneliness. It decreases the risk of living in poverty, hence increasing the ability to be socially engaged (36, 47). People with access to social services and benefits as a backup have better chances of establishing and maintaining a social network without being excessively hampered by basic livelihood concerns, such as food, housing, and medical care (20, 53), and of being socially integrated. Nyqvist et al. (25) exemplified this latter point by showing that older adults living in the Nordic regime, characterized by a high extent of state involvement in social welfare, were less dependent on their own social resources for loneliness compared to those living in regimes with a lower level of state involvement. The state may fund services that allow people to interact socially and participate in social activities. The above implies that, at the individual level, low socioeconomic status has a weaker effect on loneliness in a more generous welfare society. We therefore hypothesize that older adults with a lower socioeconomic status in countries with more generous welfare provisions are less likely to be lonely than their counterparts in countries with less generous welfare provisions (Hypothesis 2).

## Materials and methods

### Data sources

Data of the dependent variable and all individual-level predictors were obtained from the HRS family of surveys—the Survey of Health, Ageing and Retirement in Europe (SHARE) (wave 5, 2011/12) and China Health and Retirement Longitudinal Study (CHARLS) (wave 2, 2012/13). Both are representative panel surveys of the non-institutionalized older population (54), providing a unique opportunity to probe late-life loneliness in the countries studied. Harmonized datasets developed by the Gateway to Global Aging Data were also used where the comparable variables were provided (for more information, refer to www.g2aging.org). Two country-level predictors were selected based on World Development Indicators (World Bank estimate), Eurostat, and the China National Bureau of Statistics. In the study, respondents aged 50 years and older from twelve European countries (Austria, Belgium, Czech Republic, Denmark, Estonia, France, Germany, Italy, the Netherlands, Slovenia, Spain, Sweden)^1^ and China were included in the analyses. After removing individuals with missing values on any of our variables of interest (*N* = 10,879 (14.91%)), the final sample comprised 62,084 respondents. Excluded respondents were slightly older, less educated, in poorer health, and living in households with lower income.

### Dependent variable

The dependent variable of loneliness was generated based on respondents' report to the question “How often do you feel lonely?.” In the harmonized CHARLS, the question has four answer options: “rarely,” “some or a little of the time,” “occasionally or a moderate amount of time,” and “most or all of the time.” SHARE wave 5 uses a three-category response scale: “hardly ever or never,” “sometimes,” and “often.” By recoding “rarely/some or a little of the time” in CHARLS and “hardly ever or never” in SHARE to 0 and other categories to 1, a binary variable was created, contrasting older adults with loneliness and the counterparts without loneliness.

### Independent variables

#### Individual-level socioeconomic status

Respondents' household income and the individual-level socioeconomic status variable were obtained from harmonized datasets. Income measures in the Harmonized CHARLS are expressed in Chinese yuan, income measures in the SHARE in nominal euros from wave 2 (56, 57). To make this comparable across the countries studied, we generated a categorical variable (4 groups) with the highest quartile (richest group) as reference group, indicating the respondent's household income per capita position measured in quartiles estimated separately for each country.

#### Country-level income inequality and welfare generosity

The two country-level independent variables are the Gini index to measure income inequality and social expenditure as percentage of GDP to measure welfare generosity. Gini index is used as a sophisticated measurement to assess inequality across the entire society (45). It measures the extent to which the distribution of income among individuals or households within an economy deviates from a perfectly equalitarian distribution. Data used in this study are based on primary household survey data obtained from government statistics agencies and World Bank country departments. For more information and methodology, see PovcalNet (iresearch.worldbank.org/PovcalNet/index.htm). A Gini index of 0 represents perfect equality, an index of 100 implies absolute inequality – so the lower its value, the more egalitarian the distribution of income in a country.

**Figure 1:**
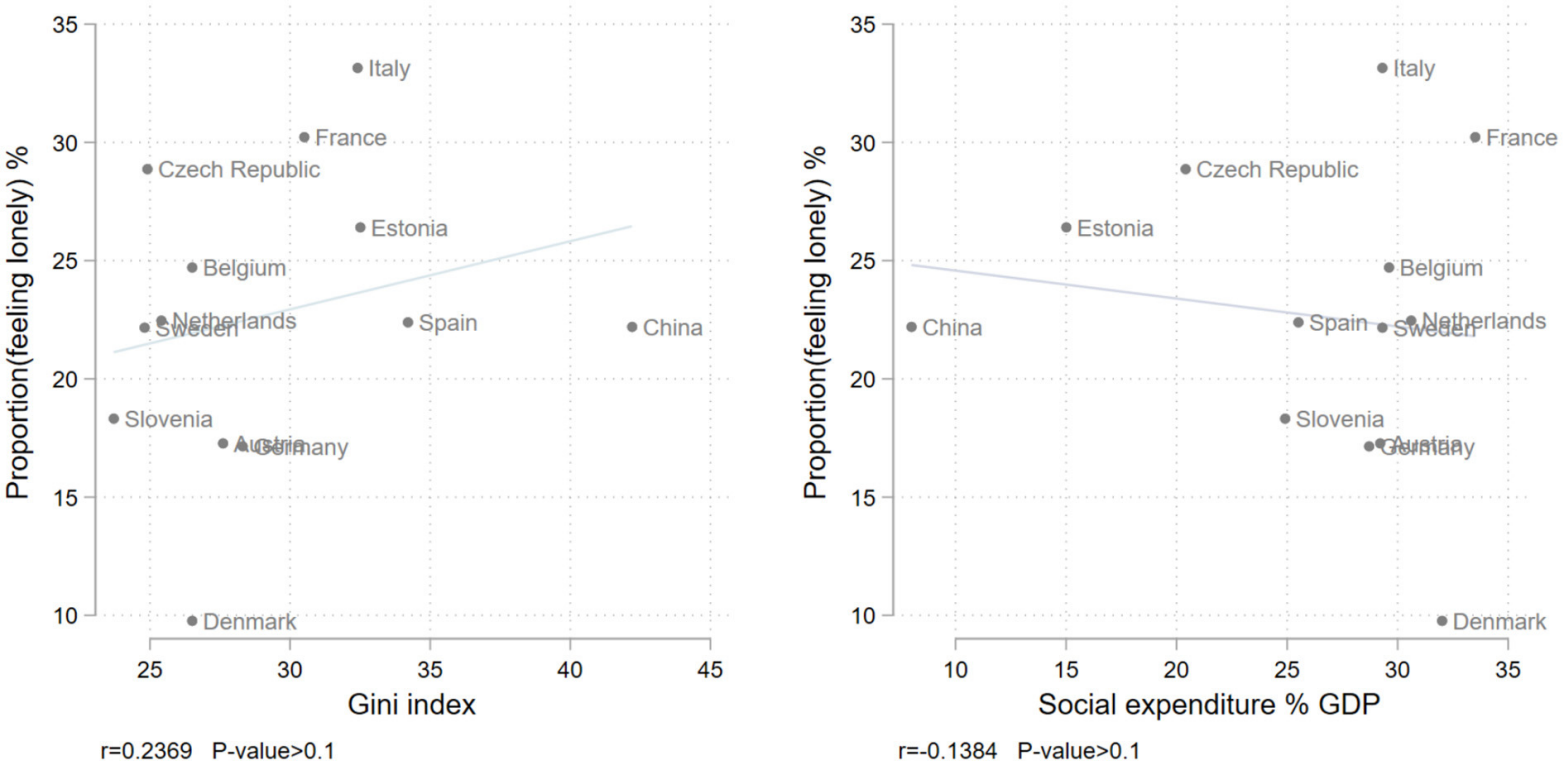
Gini index, social expenditure as percentage of GDP, and prevalence of loneliness by country.

Welfare generosity is one of the common quantitative indicators of welfare state effort and, as in this study, is mostly measured by social expenditure as percentage of GDP (49). For European countries, data of social expenditures were collected by Eurostat, comprising social benefits that consist of transfers, in cash or in kind, to households and individuals to relieve them of the burden of a defined set of risks or needs; administration costs representing the costs charged to the scheme for its management and administration; and other miscellaneous expenditures of social protection schemes (payment of property income and other)^2^. For China, the social expenditure scale based on the National Bureau of Statistics^3^ (2017) consists of government expenditures, in cash or in kind, in six domains: health, pension and elderly services, welfare for the disabled, employment protection, housing security, and other social services. Data from 2012 were used for all studied countries. Figure 1 shows income inequality and welfare generosity for these countries.

Gini index and social expenditure as percentage of GDP are correlated (*r* = −0.64, *N* = 13). With some exceptions, there is a European north-west/south-east divide with higher income inequality and less generous welfare resources in the Southern and Eastern countries. China has the highest income inequality and the lowest level of welfare generosity. Due to the considerable correlation, we examined these two country-level predictors and their interaction with individual-level income status separately.

#### Covariates

We included several covariates in the analysis that are well-known predictors of loneliness: age, gender (0 = male, 1 = female), marital status, household size, educational level, work status, health status, and child living proximity (12, 15, 58). The variable age (in years) was centered at its country sample mean. Marital status was a nominal variable with four categories: married or partnered (reference group), separated or divorced, widowed, never married. Household size was measured as the total number of persons currently residing in the household: 1 (reference group), 2, 3 or more. Educational level was recorded using the International Standard Classification of Education (59) and divided into three categories: low (less than lower secondary education), medium (upper secondary or vocational training), and high (tertiary education, reference group). Work status was generated from the response to whether (= 1) or not (= 0) working for pay last year (CHARLS), and since the last interview or in the last four weeks (SHARE). Subjective health was assessed by asking respondents to report their health status, with response options from 1 “excellent” to 5 “poor.” Due to a low proportion of respondents answering “excellent,” we combined “excellent” with “very good.” Limitations in instrumental activities of daily living (IADLs) were assessed by asking respondents whether they experience any difficulty performing six tasks: managing money, taking medications, shopping for groceries, preparing a meal, cleaning the house, making phone calls. An IADLs summary score adjusted by the number of IADL questions with missing values was used in the analyses. Child living proximity variable was categorized into “no child” (reference group), “live with at least one child,” “at least one child lives nearby,” or “all children live far.” Table 1 shows the characteristics of the study population.

**Table 1:** Characteristics of the study population.

	**Austria**	**Belgium**	**China**	**Czech Rep**.	**Denmark**	**Estonia**	**France**	**Germany**	**Italy**	**Netherlands**	**Slovenia**	**Spain**	**Sweden**	**Overall**
Age (mean)	66.85	65.45	61.99	66.55	64.88	67.94	67.14	64.46	66.38	65.68	66.51	67.70	67.99	65.84
Female (%)	57.80	54.74	49.91	58.62	53.43	61.23	57.04	52.37	54.45	54.75	56.86	53.77	53.30	57.80
Marital status (%)
Married/partnered	65.71	71.80	87.19	70.05	78.05	67.16	68.54	79.94	79.46	79.64	74.35	80.01	77.69	76.17
Divorced/separated	11.78	10.65	1.22	10.69	7.95	9.15	8.88	6.91	2.88	6.86	4.26	2.90	8.55	6.78
Widowed	15.68	12.57	10.80	17.47	10.00	18.69	16.63	9.07	11.89	10.08	16.73	12.02	8.72	12.93
Never married	6.83	4.98	0.79	1.79	3.99	4.99	5.96	4.08	5.77	3.43	4.66	5.07	5.04	4.12
Education (%)
High	26.16	33.79	1.30	13.05	42.45	21.60	21.46	29.61	8.28	27.81	17.38	10.56	30.82	20.21
Medium	61.68	48.69	10.03	74.39	47.44	72.95	43.89	68.68	44.91	61.58	72.73	31.39	47.61	49.66
Low	12.16	17.52	88.67	12.56	10.11	5.45	34.65	1.71	46.81	10.62	9.89	58.05	21.57	30.13
Working (%)	26.70	33.57	66.72	32.30	48.52	37.62	30.87	43.50	25.45	37.74	18.97	26.67	43.65	38.77
Subjective health (%)
Very good	33.07	28.66	9.86	16.88	54.69	5.14	20.60	20.52	21.25	28.04	17.84	19.17	43.84	22.81
Good	35.52	44.17	14.02	38.49	23.34	23.13	42.47	39.42	36.10	43.49	44.68	39.27	32.39	33.55
Fair	24.71	21.77	53.90	30.57	16.91	50.31	26.07	30.35	30.28	23.89	25.62	28.43	18.49	31.61
Poor	6.71	5.40	22.22	14.06	5.07	21.41	10.86	9.72	12.37	4.58	11.86	13.13	5.28	12.03
IADLs (mean)	0.03	0.04	0.09	0.03	0.03	0.05	0.04	0.03	0.04	0.02	0.03	0.05	0.02	0.04
Household size (%)
1	30.42	24.17	4.94	24.32	21.79	26.37	27.83	17.93	15.36	20.67	18.49	13.51	22.93	19.51
2	53.26	55.56	35.38	56.96	65.08	56.88	56.99	63.56	46.49	64.95	50.73	52.85	69.47	54.61
3+	16.32	20.26	59.68	18.72	13.13	16.76	15.18	18.52	38.16	14.38	30.78	33.63	7.60	25.88
Children (%)
Childless	12.39	12.19	1.88	4.32	8.22	8.89	10.45	11.81	12.46	10.57	6.77	10.56	7.19	8.63
Lives with at least one child	15.12	17.92	45.10	16.78	11.37	15.45	11.11	18.30	30.65	13.07	29.60	28.07	8.84	21.84
At least one child lives nearby	52.86	51.65	41.76	60.48	45.42	46.79	40.46	47.41	38.61	51.78	55.11	45.87	52.22	47.97
All children live far	19.63	18.24	11.26	18.42	35.00	28.86	37.98	22.48	18.28	24.58	8.53	15.50	31.75	21.57
*N*	*3,922*	*5,241*	*8,785*	*5,256*	*3,809*	*5,287*	*4,078*	*5,444*	*4,382*	*3,909*	*2,791*	*5,483*	*4,186*	*62,573*

The italic values indicate the number of observations.

#### Analytical approach

We first descriptively explored the association between an individual's loneliness and household income on a micro level per country (Table 2) and the correlations between country-level factors and prevalence of loneliness (Figure 1). Next, logistic country fixed effect models were built to examine the risk of feeling lonely by the individual's household income status, and how the potential influence of individual-level income varied in two macro contexts measured as Gini index and social expenditure (Table 3). In the fixed effect models the data from different countries were pooled, and the model specification included distinct country intercepts estimated as the coefficients on country dummies (Denmark is the reference group). The country dummies are controls and do not reflect a test of the research hypotheses. As we expected income inequality and welfare generosity to be a trigger and a buffer against the risk of loneliness among socioeconomically disadvantaged older adults, the cross-level interaction of the individual-level and the country-level variables were included to operationalize the moderating effect of macro contexts on the association between individual socioeconomic status and late-life loneliness. In doing so, the individual effects of economic status are allowed to differ between countries (60).

**Table 2:** Descriptive statistics for the sample of older adults (50+) by country.

	***N***	**Feel**	**Household income**		***p* ^a^**
		**lonely (%)**	**per capita**		
			**Mean**	**SD**	
Austria	*3,877*	17.28	17,306	11,128	
Belgium	*5,150*	24.72	26,012	30,120	***
China^b^	*8,785*	22.21	9,766	24,240	***
Czech Republic	*5,177*	28.88	5,330	3,393	+
Denmark	*3,774*	9.78	24,976	13,789	***
Estonia	*5,266*	26.41	5,603	5,191	***
France	*4,049*	30.23	23,527	116,363	**
Germany	*5,386*	17.16	18,930	13,303	***
Italy	*4,361*	33.16	11,241	22,174	*
Netherlands	*3,858*	22.47	22,372	21,904	**
Slovenia	*2,788*	18.33	7,465	7,988	**
Spain	*5,455*	22.40	9,711	8,724	*
Sweden	*4,158*	22.17	28,800	15,899	***

+ p < 0.10,

* p < 0.05,

** p < 0.01,

*** p < 0.001.

a Pearson Chi-square test is used to determine whether there is a statistically significant difference in the likelihood of feeling lonely by quartile of household income per capita groups.

b Income measures for the 12 European countries are expressed in euros, for China in yuan. The average yuan-to-euro exchange rate was 0.1233 at the time of the survey.

c The italic values indicate the number of observations.

**Table 3:** Fixed effect models: main effect and interaction effects on the occurrence of feeling lonely for older adults (*N* = 62,084).

	**Model 1**	**Model 2**	**Model 3**
**Household income status (ref.: highest 25%)**			
Upper 25%	0.060*	−0.321	0.232*
Lower 25%	0.124***	−0.439**	0.461***
Lowest 25%	0.146***	−0.372*	0.353***
**Household income & Income inequality**			
Upper 25% × Gini		0.013*	
Lower 25% × Gini		0.018***	
Lowest 25% × Gini		0.017**	
**Household income &Welfare generosity**			
Upper 25% × social expenditure			−0.007
Lower 25% × social expenditure			−0.014***
Lowest 25% × social expenditure			−0.009*
Pseudo R-squared	0.129	0.130	0.130
AIC	58326.2	58317.8	58317.1
BIC	58624.4	58643.1	58642.4

* p < 0.05,

** p < 0.01,

*** p < 0.001.

Coefficients omitted for control variables: age, gender, marital status, education, employment, self-rated health, functional limitations, household size, child and living arrangement, and country.

Compared with the multilevel approach, which is a more straightforward strategy to examine multilevel hypotheses (61), the FE method can be applied with small samples at a higher level, avoiding the omitted variable bias (58), such as influence of cultural norms on loneliness. One limitation of FE models is that estimates of the fixed parameters may be imprecise, particularly if associated with country-level factors, but they are generally unbiased (62–64).

## Results

The descriptive statistics by country in Table 2 show that the highest prevalence of loneliness among older adults was in Italy, France, the Czech Republic, and Estonia (26–33%). Denmark had the lowest proportion of lonely older adults (10%), followed by Austria and Germany (17%). In the remaining countries, including China, the prevalence of loneliness ranged between 22 and 25%. Sweden, Belgium, Denmark, France, and the Netherlands had the highest household income per capita (above 20,000 euros), the Czech Republic, Estonia, Slovenia, Spain, and China the lowest (below 10,000 euros and 9,766 Chinese yuan). Bivariate analysis showed a significant negative association between individuals' household income and loneliness in each country, except for Austria. Correlations between prevalence of loneliness and the two country-level economic factors (Gini index and social expenditure) were low and not statistically significant (see Pearson correlation coefficient *r* and *p*-value in Figure 1).

Table 3 shows the estimated coefficients from logistic country fixed effect (FE) models for the impact of individual-level household income and its interaction effect with country-level Gini index and social expenditure on loneliness. Model 1 included the estimates of individual-level household income. The lower the household income, the greater the likelihood of feeling lonely. Compared to those in the highest quartile of income, the odds ratio for older adults in the lowest quartile was 1.16 (95% CI: 1.09–1.23). The contextual moderating effects at the macro level were estimated in models 2–3. In both macro contexts—income inequality measured as Gini index and welfare generosity measured as social expenditure as percentage of GDP—we found a significant interaction effect with the individuals' household income in the expected direction, supporting our two hypotheses: older adults with lower socioeconomic status in less income-equal societies are more likely to be lonely than their counterparts in more income-equal societies (Hypothesis 1), and older adults with lower socioeconomic status in societies with more generous welfare expenditures are less likely to be lonely than their counterparts in less generous societies (Hypothesis 2).

**Figure 2:**
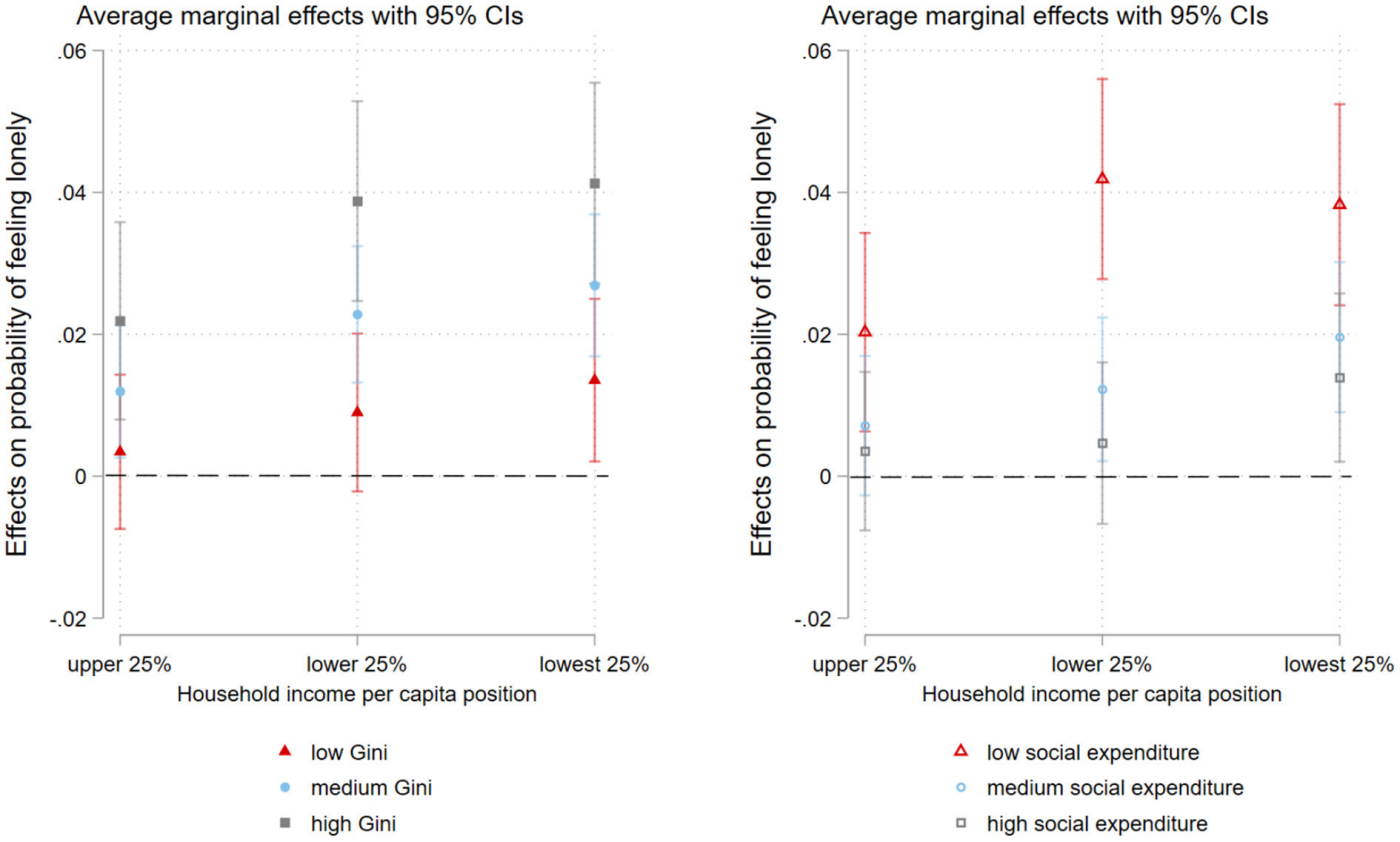
Average marginal effects of individual-level household income status (compared to the highest 25%) on probability of feeling lonely in different macro contexts.

Figure 2 visualizes these associations by showing the average marginal effects of individual-level household income status on loneliness in various income-unequal conditions and levels of welfare generosity. The left graph in Figure 2 shows that in the most inequitable context, the likelihood of loneliness was most pronounced among older adults who were in the lowest income quartile compared to their age peers in the higher income quartiles, whereas in a more income-equal society the effect of individual-level household income had less impact on likelihood of loneliness. For the interaction effect of social expenditure and household income on loneliness, the right graph in Figure 2 shows that for older adults living in a country with less generous welfare resources, household income was closely associated with risk of loneliness. However, for the lowest income group in societies with lower social expenditures, the risk of loneliness was not as high as for the low-income group.

## Discussion

The aim of our study was to examine whether country-level income inequality and welfare generosity moderate the effect of individual-level socioeconomic status on late-life loneliness. Our results showed a significant interaction effect between Gini index of income inequality at the macro level and household income at the micro level on loneliness, which supports our first hypothesis. This finding is in line with Du, King and Chi (42), who found an interplay between countries' income inequality and individual income, and individuals' subjective wellbeing and psychological distress. Our results also show a significant interaction effect on loneliness between welfare states' social expenditures and individual household income, supporting our second hypothesis. This finding confirms previous research indicating that lower socioeconomic classes are more strongly affected by the level of state involvement in welfare provisions (43, 64, 65).

There is, however, a difference in the impact of income inequality and welfare states' social expenditures for the low and lowest socioeconomic classes. When there is more income inequality in a country, the higher likelihood of loneliness is nearly equal for the low and lowest socioeconomic classes, whereas with lower state social expenditures the likelihood of loneliness is significantly higher for the low socioeconomic class than for the lowest class. One possible explanation for the latter difference could be related to the selective means-tested or residual model, which has been criticized for trapping low-income people in relative poverty because in that welfare model their savings result in a loss of opportunities to receive welfare benefits from the government (66). In societies with the least generous welfare resources, the key focus lies on improving the living conditions of the poorest, enabling them to get access to food, sanitation, and housing (45, 53). For example, Estonia, which has a relatively low level of welfare generosity, applies the basic welfare model to prevent people from falling into extreme poverty (67). A similar situation exists in China, which has the least generous welfare system of all the countries studied (see Figure 1). Although China has significantly developed and reformed its social welfare and elder care, the government has continued to use the residual welfare model, which provides the poorest older adults in need with basic benefits for food, clothing, housing, medical treatment, and funeral services (68, 69). If the welfare system does not target the low socioeconomic group they do not receive welfare resources, their feelings of relative deprivation are likely to rise, and the risk of loneliness will increase.

Our study sheds light on the importance of more equalitarian income distribution and more generous welfare provision spending in preventing or reducing loneliness among older adults of low socioeconomic status. Individuals are embedded in larger material and societal contexts that shape the actual or perceived quality of living conditions and create opportunities for social integration (6, 20). There is a need not only to target individuals at risk, but also to identify contextual aspects of societies in terms of loneliness prevention and alleviation (70). In light of this study, anti-poverty measures, income maintenance programs, and comprehensive pension systems are highly recommended, as is ensuring equity and fairness in provision. National and local governments could also offer free or low-cost services to older adults of low socioeconomic status, allowing them to interact socially and participate in social activities.

## Data availability statement

The Survey of Health, Ageing and Retirement in Europe (SHARE) is centrally coordinated at the Munich Centre for the Economics of Aging (MEA). The China Health and Retirement Longitudinal Study (CHARLS) is conducted and supported by Peking University, the National Natural Science Foundation of China, the Behavioural and Social Research Division of the National Institute on Aging and the World Bank. The harmonized datasets of both surveys were publicly released through the website of the Gateway to Global Aging Data (www.g2aging.org).

## Ethics statement

Ethical review and approval and written informed consent for participation were not required for this study because this study is based solely on publicly available secondary data. It is in accordance with the national legislation and the institutional requirements.

## Author contributions

The research idea and design were conceived by JW. Data collection and cleaning were performed by JZ and JW. Statistical analyses were conducted by JZ under the cooperation and discussion with JW and TF. JW took the lead in writing the manuscript. TF and JZ provided feedback and revised several versions of the manuscript. All authors have read and agreed to the published version of the manuscript.

## Funding

JW acknowledges funding support from the Donation Funds at the University of Gothenburg for Teachers' Research and Travel for Scientific Purposes in 2022 (GU 2022/18).

## Conflict of interest

The authors declare that the research was conducted in the absence of any commercial or financial relationships that could be construed as a potential conflict of interest.

## Publisher's note

All claims expressed in this article are solely those of the authors and do not necessarily represent those of their affiliated organizations, or those of the publisher, the editors and the reviewers. Any product that may be evaluated in this article, or claim that may be made by its manufacturer, is not guaranteed or endorsed by the publisher.

## Footnotes

^1^Because Israel and Switzerland are not EU countries, and Luxembourg is an extremely small and wealthy country with only 62.9% respondents with citizenship status (55), we dropped these country samples.

^2^Source: Eurostat, Expenditure on social protection, https://ec.europa.eu/eurostat/tgm/table.do?tab=table&init=1&plugin=1&language=en&pcode=tps00098.

^3^Source: National Bureau of Statistics, Comparative Analysis of Social Security Expenditure between China and EU (in Chinese), http://www.stats.gov.cn/tjzs/tjsj/tjcb/dysj/201709/t20170929_1539209.html.
